# Hands-free continuous carotid Doppler ultrasound for detection of the pulse during cardiac arrest in a porcine model

**DOI:** 10.1016/j.resplu.2023.100412

**Published:** 2023-06-20

**Authors:** Bjørn Ove Faldaas, Erik Waage Nielsen, Benjamin Stage Storm, Knut Tore Lappegård, Ole-Jakob How, Bent Aksel Nilsen, Gabriel Kiss, Eirik Skogvoll, Hans Torp, Charlotte Ingul

**Affiliations:** a Department of Circulation and Medical Imaging, Norwegian University of Science and Technology (NTNU), Trondheim, Norway; b Faculty of Nursing and Health Sciences, Nord University, Bodø, Norway; c Department of Clinical Medicine, Faculty of Health Sciences, UiT the Arctic University of Norway, Tromsø, Norway; d Department of Anesthesia, Surgical Clinic, Nordland Hospital Trust, Bodø, Norway; e Department of Immunology, Oslo University Hospital, University of Oslo, Oslo, Norway; f Research Laboratory, Nordland Hospital Trust, Bodø, Norway; g Department of Medicine, Nordland Hospital Trust, Bodø, Norway; h Department of Medical Biology, Faculty of Health Sciences, UiT the Arctic University of Norway, Tromsø, Norway; i Department of Computer Science (IDI), Faculty of Information Technology and Electrical Engineering, Norwegian University of Science and Technology (NTNU), Trondheim, Norway; j Clinic of Anesthesia and Intensive Care Medicine, St Olav University Hospital, Trondheim, Norway

**Keywords:** Doppler ultrasound, Experimental model, Carotid artery blood flow, Cardiac arrest monitor, PEA, Ventricular fibrillation

## Abstract

**Background/Purpose:**

Pulse palpation is an unreliable method for diagnosing cardiac arrest. To address this limitation, continuous hemodynamic monitoring may be a viable solution. Therefore, we developed a novel, hands-free Doppler system, RescueDoppler, to detect the pulse continuously in the carotid artery.

**Methods:**

In twelve pigs, we evaluated RescueDoppleŕs potential to measure blood flow velocity in three situations where pulse palpation of the carotid artery was insufficient: (1) systolic blood pressure below 60 mmHg, (2) ventricular fibrillation (VF) and (3) pulseless electrical activity (PEA). (1) Low blood pressure was induced using a Fogarty balloon catheter to occlude the inferior vena cava. (2) An implantable cardioverter-defibrillator induced VF. (3) Myocardial infarction after microembolization of the left coronary artery caused True-PEA. Invasive blood pressure was measured in the contralateral carotid artery. Time-averaged blood flow velocity (TAV) in the carotid artery was related to mean arterial pressure (MAP) in a linear mixed model.

**Results:**

RescueDoppler identified pulsatile blood flow in 41/41 events with systolic blood pressure below 60 mmHg, with lowest blood pressure of 19 mmHg. In addition the absence of spontaneous circulation was identified in 21/21 VF events and true PEA in 2/2 events. The intraclass correlation coefficient within animals for TAV and MAP was 0.94 (95% CI. 0.85–0.98).

**Conclusions:**

In a porcine model, RescueDoppler reliably identified pulsative blood flow with blood pressures below 60 mmHg. During VF and PEA, circulatory arrest was rapidly and accurately demonstrated. RescueDoppler could potentially replace unreliable pulse palpation during cardiac arrest and cardiopulmonary resuscitation.

## Highlights

• Continuous circulation check with Doppler ultrasound.
• Detection of carotid blood velocity in a hypotension animal model.
• Hands-free carotid Doppler detects circulation after defibrillation.
• Detection of ROSC with hands-free Doppler ultrasound.
• True-PEA identified with hands-free Doppler ultrasound.
• Continuous circulation check with Doppler ultrasound.

## Introduction

Sudden cardiac arrest is an abrupt cessation of cardiac pump function, resulting in the absence of signs of circulation, including the absence of a pulse. During advanced cardiopulmonary resuscitation (CPR^1^), chest compressions are paused briefly to assess the cardiac rhythm by electrocardiogram (ECG) and the return of spontaneous circulation (ROSC) by manual carotid pulse palpation.^1^ Pulse palpation has severe limitations and is neither a rapid nor a reliable method.^2^, ^3^, ^4^, ^5^ During CPR, 45% of the healthcare providers were unable to detect central pulse accurately^2^, ^6^ despite a systolic radial artery pressure of above 80 mmHg.^3^ In addition, pulseless electrical activity (PEA) was misdiagnosed in 32% of cardiac arrests, where the patient had blood circulation.^7^

Since invasive blood pressure seldom is used during cardiac arrest, a surrogate marker for ROSC is used, such as end-tidal carbon dioxide (ETCO2).^1^, ^8^, ^9^ However, the accuracy of ETCO2 is influenced by, e.g. ventilation and medications.^8^, ^9^, ^10^

PEA is increasingly and particularly prevalent in both in-hospital (IHCA) and out-of-hospital (OHCA) cardiac arrest.^11^, ^12^ Recent research from our research group at NTNU^13^, ^14^ shows that PEA behaves very differently in terms of developing ROSC, depending on the preceding rhythm (primary, or secondary to temporary ROSC, ventricular fibrillation (VF), ventricular tachycardia (VT), or asystole). Thus, incorrect decisions about the presence or absence of a carotid pulse, both initially and when evaluating the response to treatment, may deprive the patient of an individualized approach. This may lead to both under- and overtreatment, the latter for instance by schematic applying potentially harmful chest compressions and medications like adrenaline.^15^ The European Resuscitation Council Guidelines' emphasise high quality chest compressions with minimal interruption.^1^, ^16^ The most common reason for interrupting chest compression for more than 10 seconds was rhythm/pulse checks in 52% of 206 IHCA and OHCA.^17^ The use of ultrasound for pulse checks has increased but might also cause additional or prolonged interruptions of chest compressions and requires a skilled operator.^1^, ^18^, ^19^, ^20^

Therefore, we aimed to evaluate the ability to detect blood flow with a novel, automated, hands-free pulsed wave Doppler system, RescueDoppler. We evaluated RescueDoppler in three clinical situations.

Our hypothesis was whether Doppler could identify carotid pulse in situations where blood pressure is below 60 mmHg and pulse palpation is unreliable,^3^, ^21^ and identify the absence of pulse in situations such as VF and true PEA where circulation has ceased.

## Methods

### RescueDoppler system

The RescueDoppler is a pulsed wave Doppler system consisting of a custom-made carotid ultrasound Doppler probe, a scanner (Manus EIM-A produced by Aurotech Ultrasound AS), and a laptop running Matlab (Matlab® R2021a) program as the user interface, and a real-time display (Supplemental Fig. 1), similar to the NeoDoppler system.^22^ The RescueDoppler system was tested and complied with the thermal and acoustic requirements of international standard IEC60601-2-37 for clinical research for non-scanned modalities.

The RescueDoppler system involved two transducers fitted to a 3D-printed transducer casing with fixed angle ±30° (Supplemental Fig. 1). The aperture of each transducer was unfocused with a dimension of 30 × 6 mm and a central frequency of 4 MHz. This multirange technology used digital signal processing and 32 depth ranges distributed equally from 8 to 45 mm.

The system displayed the depth ranges as colour M-mode and Doppler spectrogram of the selected range and sample volume (Supplemental Fig. 2). The maximum velocity curve was automatically traced from the spectrogram. Doppler variables, peak systolic velocity, end-diastolic velocity, and time-averaged blood flow velocity (TAV) were calculated for each heartbeat. TAV was calculated from each transducer, and angle is estimated by a simple geometrical formula, based on the two TAV values and the fixed angle between the transducers. Values for resistive index, pulsatility index, and heart rate are not affected by inclination angle. Angle correction was performed after the experiments.

### Animals

Twelve pigs (sus scrofa domesticus) were included (mean weight 30 ± 3 kg). The study was approved by the Norwegian Animal Research Authority (FOTS-ID 25415) and performed by the ARRIVE guidelines.^23^

### Animal retrieval, preparation, and anaesthesia

At the farm, the animals were sedated with intramuscular 1000 mg of ketamine (Ketalar, Pfizer AS, Norway), 1 mg of atropine (G.L. Pharma GmbH, Lannach, Austria), and 5 mg of midazolam (B. Braun Melsungen AG, Germany) before being transported the laboratory. A 20-G peripheral venous catheter (BD Venflon™ Pro Safety Needle, Eysins, Switzerland) was inserted into both ears. Anesthesia was induced with intravenous (iv) midazolam and morphine (G.L. Pharma GmbH, Lannach, Austria) and maintained with iv morphine, midazolam, and thiopental (Pentocur Abcur AB, Sweden). Methods for sedation and anaesthesia were conducted as described in detail by Storm et al.^24^ The animals were ventilated using a GE Engstøm Carestation Ventilator (GE Healthcare) with 21% FiO_2_, a tidal volume of 15–20 mL/kg, a respiratory rate of 13 –16 breaths per minute, and zero positive end-expiratory pressure. End-tidal CO2 (ETCO2) was continuously monitored, and minute ventilation was adjusted to maintain a normal pH and normocapnia. The pigs were kept normothermic (38.5–39.0 °C).

### Instrumentation

The left external jugular vein and left internal carotid artery were identified using a Sonosite S ultrasound machine with an L38 linear transducer (Fujifilm Sonosite, Bothell, WA).

Ultrasound-guided Seldinger's technique was used to insert an 8 Fr Avanti + Sheath introducer (Cordis, Santa Clara, CA) into the left external jugular vein and a 4 Fr, 8 cm leader arterial catheter (Vygon Ltd., Swindon, UK) into the left internal carotid artery. An open surgical technique was used if more than one attempt was necessary.

### Monitoring and data recording

A TruWave pressure monitoring transducer kit (Edwards Lifesciences Corporation, Irvine, CA) was connected to an arterial cannula, and 500 mL of pressurized Ringer’s acetate (Baxter AS, Norway) and 1250 IE heparin (LEO Pharma AS, Norway) was added to prevent catheter clotting. The flow rate across the flush device was 3 ± 1 mL/hr. Monitoring: continuous 5-lead ECG, continuous invasive arterial- and central venous pressures, ETCO2, peripheral oxygen saturation (SPO2), and urinary bladder temperature. These data were recorded by Phillips IntelliVue Patient Monitor MP70 (Philips Medizin Systeme Boeblingen GmbH). The common carotid artery (CCA) on the contralateral side of the arterial catheter was identified by vascular ultrasound (GE Vivid S7 Pro) linear probe. The RescueDoppler probe was positioned over the CCA to ensure optimal signal recording and fixed with a elastic self-adhering bandage around the neck. An overview of the research setup is presented in Supplemental Fig. 1. For precise time synchronization between the RescueDoppler recordings and other physiological recordings, an electrical pulse-generator (impulse every 6 seconds) was connected to the RescueDoppler computer and a pressure transducer on the MP70 monitor. The pulses were timestamped and recorded by the RescueDoppler computer and the MP70 monitor. These signals were used for precise post-processing data synchronization. Data from the IntelliVue monitor and the ventilator were transferred to the RescueDoppler laptop via two additional serial ports. A custom Matlab program was designed to synchronize the traces from and calculate the derived parameters (Supplemental Table 1).

### Vena cava occlusion model

**Fig. 1:**
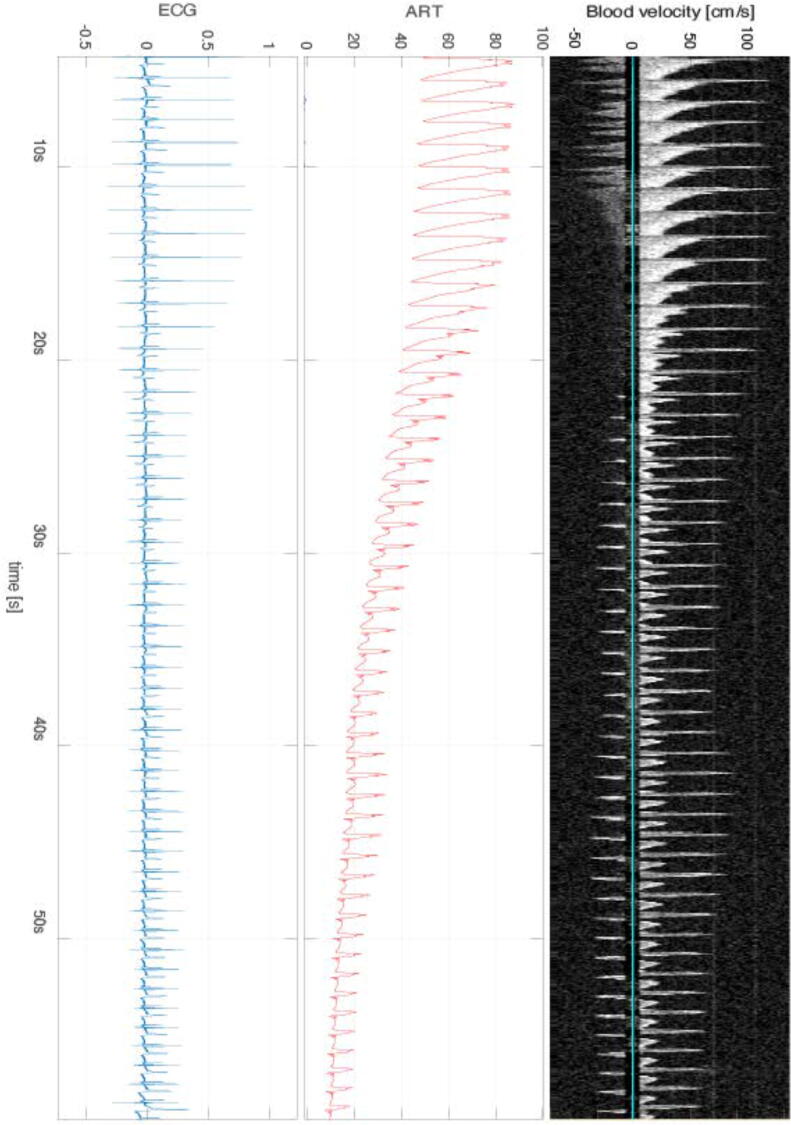
Vena cava occlusion sequence from base to lowest systolic blood pressure. The top line shows the carotid Doppler spectrogram, cm/sec. The middle line shows arterial pressure in the carotid, mmHg. The lower image shows an electrocardiogram. The figure shows a vena cava occlusion sequence from the start to the lowest blood pressure before the cessation of occlusion. Blood pressure drops gradually from around 100 to 25 mmHg systolic pressure. Doppler velocity follows pulsative blood pressure beat by beat. Little reduction in peak flow is seen, and negative flow is visible from systolic pressure 80 mmHg down to the lowest value.

Hypotension was induced by occlusion of the inferior vena cava (VCO) using a 6 Fr Edwards Fogarty Arterial Embolectomy Catheter (Edwards Lifesciences Corporation). The catheter was brought forward to the inferior vena cava and placed intrathoracically above the diaphragm (Supplemental Fig. 1). We then occluded the inferior vena cava by inflating the catheter cuff with 2 mL of normal saline. This resulted in a rapid fall in blood pressure (Fig. 1). The occlusion was terminated at the lowest possible systolic blood pressure below 60 mmHg. Ventilation was paused during the sequences, from inflation to deflation of the catheter cuff.

### VF model

We induced VF with an implantable cardioverter-defibrillator (ICD) (St. Jude Medial Ellipse DR Model CD2377-36C, Merlin Patient Care System, Abbott, formerly St. Jude Medical Inc) (Supplemental Fig. 1). The ICD lead was inserted through the external jugular vein to the right ventricle and placed in the apex using echocardiographic guidance (GE Vivid S7 Pro, 3S probe), and the device was placed subcutaneously on the upper right side of the chest. VF was induced using the DC Fibber induction method with the St. Jude Medical software.^25^ A 7.5-volt direct current was delivered to the myocardium for 2 seconds. Defibrillation (30 joules) was automatically delivered by the ICD after a maximum of 60 seconds of untreated VF. The interval between two VF episodes was 5 minutes. FiO_2_ was increased to 100% before and after each sequence. Subsequent defibrillation converted all VF sequences to sinus rhythm and ROSC.

### Myocardial infarction model

Infarction (MI) was induced by left coronary microembolization. The main trunk of the left coronary artery was catheterized with a 4 Fr angiography catheter under fluoroscopic guidance. Microembolization was performed using 50 μm polystyrene microspheres (Chromosphere, Thermo Scientific™) dissolved in 0.9% sodium chloride and 0.01% Tween 20. The model is previously described by Stenberg et al.^26^ We administered polystyrene microspheres (50–150 mg) to induce a significant impairment in contractile function, with the objective of decreasing blood pressure to a systolic pressure of 60 mmHg or lower.

### Statistics

Descriptive statistics include basic animal data, blood pressure, and Doppler velocity measurements at baseline and VCO. The sample size for the model was based on similar studies.^27^, ^28^ Each pig underwent repeated sequences of VCO or VF. We investigated the ability of RescueDoppler to track blood pressure during the VCO sequences by fitting a linear mixed model with TAV as the outcome variable to MAP up to the second degree as the fixed predictor variable. We entered the animal identity and sequences nested within animals as random intercepts, and MAP up to the second degree as random slopes within animals. The variances ascribed to animals and sequences within animals in this two-level hierarchical model were quantified as intraclass correlation coefficients (ICC). All statistical analyses were performed in IBM SPSS Statistics version 27.0.1.0. Matlab was used to display the data and for post-data analysis. Stata version 17 used the “mixed” procedure (StataCorp, College Station, Texas, USA) to estimate the linear mixed-effects model.

## Results

Twelve pigs (male *n* = 11) were included in the present study VCO model *n* = 7; VF model *n* = 4, MI model *n* = 7 (Table 1). The Doppler recorded flow continuously for a mean duration of 26 minutes (range 18–46 min) in the VCO model and 48 minutes (range 28–71 min) in the VF model. MI model was performed after the VCO model. Data from one VCO animal were excluded due to technical failure.

**Table 1:** Baseline characteristics of the animals for vena cava occlusion (VCO) model and Ventricular fibrillation (VF) model.

VCO model, *n* = 7	Range	Minimum	Maximum	Mean	Male (Female)
Carotid diameter (mm)	13	37	50	43	
Carotid depth (mm)	60	19	25	22	
Weight (kg)	4	28	32	30	
Sex					6 (1)

VF model, *n* = 4	Range	Minimum	Maximum	Mean	Male (Female)

Carotid diameter (mm)	2	48	50	50	
Carotid depth (mm)	200	20	40	28	
Weight (kg)	6	27	33	30	
Sex					4 (0)

### VCO model

**Fig. 2:**
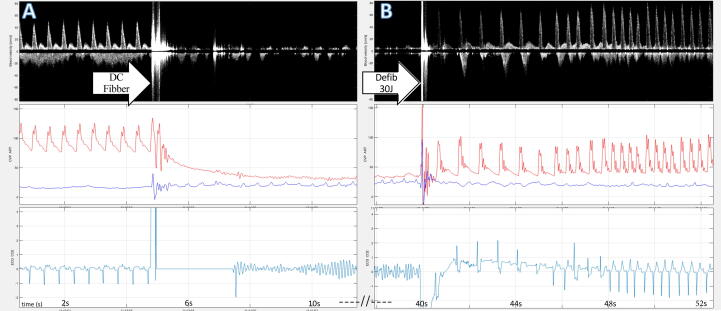
Ventricular fibrillation model. Panel A and B are consecutive. Top line: RescueDoppler velocity curve. Middle line: Arterial pressure, red curve, and central venous pressure, blue curve. Bottom line: electrocardiogram. The figure shows an example where circulation ceases. Cessation of pulsative velocity occurs simultaneously with the fall in blood pressure. After defibrillation, return of spontaneous circulation occurs simultaneously with return of pulsative arterial pressure and carotid Doppler velocity. Top line: Inducton of ventricular fibrillation appears as white noise on the doppler curve before pulsative blood flow ceases. Middle line: sudden fall in blood pressure and loss of pulsative pressure. Bottom line: Sinus rhythm before induction, followed by a short period of signal loss before ventricular fibrillation.

VCO sequences (*n* = 41) from seven pigs were included (Table 2). Each sequence lasted 50 seconds (range 30–69 sec) from inflation to deflation. VCO systolic pressures: mean 33 mmHg and minimum 19 mmHg (Table 2). The lowest peak systolic velocity was a mean of 102 cm/sec and a minimum of 74 cm/sec (Table 2). An illustration of the VCO model is presented in Fig. 2.

**Table 2:** Baseline and low blood pressure variables for vena cava occlusion sequences.

	*n* = 41				
*Baseline variables	Range	Minimum	Maximum	Mean	Std. Deviation
Peak Systolic Pressure (mmHg)	47	86	133	111	12.9
Mean Arterial Pressure (mmHg)	40	66	106	86	11.4
Peak Diastolic Pressure (mmHg)	35	45	81	66	9.6
Central Venus Pressure (mmHg)	21	−4	16	6	5.5
Peak Systolic Velocity (cm/s)	67	74	142	102	16.7
Time-average velocity (cm/s)	41	23	64	37	10.4
End-diastolic velocity (cm/s)	68	−40	29	10	19.0

**Values of variables at the low blood pressure	*n* = 41				
	Range	Minimum	Maximum	Mean	Std. Deviation

Peak Systolic Pressure (mmHg)	27	19	46	33	7.8
Mean Arterial Pressure (mmHg)	21	12	33	23	5.5
Peak Diastolic Pressure (mmHg)	19	8	27	18	5.4
Central Venus Pressure (mmHg)	21	−5	16	4	6.2
Peak Systolic Velocity (cm/s)	52	46	98	73	14.7
Time-average velocity (cm/s)	24	4	27	16	4.9
End-diastolic velocity (cm/s)	48	−57	−9	−29	13.2

* Baseline variables recorded approximately 10 seconds prior to the vena cava occlusion sequence.

** Low values of variables are measured at the lowest systolic blood pressure before deflation of the Fogarty catheter cuff. Std. Deviation = Standard Deviation.

### VF model

VF sequences (*n* = 21) from four pigs were included. The model is illustrated in Fig. 2.

Cessation of circulation was immediately confirmed by the absence of invasive carotid blood pressure and VF on the ECG. All 21 VF sequences were recorded and confirmed in real-time with the absence of blood pressure and Doppler pulsative blood flow (Fig. 2).

### TAV and MAP

**Fig. 3:**
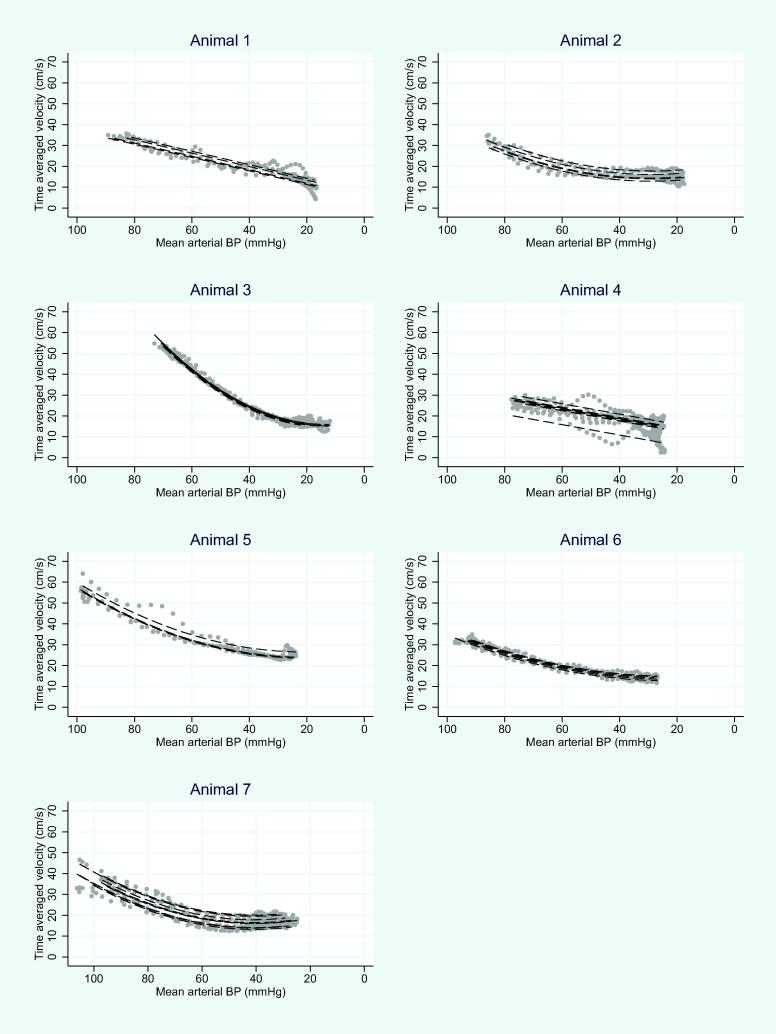
Seven scatter plots are presented, one for each animal, depicting the relationship between Time-Averaged Velocity (TAV) and Mean Arterial Pressure (MAP) during Vena Cava Occlusion (VCO). The Y-axis represents TAV, and the X-axis represents MAP, with identical scales for all plots. The X-axis is transposed to demonstrate the time-based progression of VCO. The VCO sequence was captured and recorded every second, from its initiation to completion. The obtained data is visually represented in the figure with gray dots. The statistical analysis utilized a second-degree random slope for MAP within each animal and individual intercepts for each sequence, indicated by black dashed lines. The Intraclass Correlation Coefficient (ICC) between TAV and MAP was determined to be 0.88 (95% CI 0.70–0.96) for the animals as a whole, and 0.94 (95% CI 0.85–0.98) when considering within-animal variance.

Fig. 3 shows scatter plots for each animals’ sequence, respectively, overlaid predicted values obtained from the linear mixed effects model. Within each animal and sequence, TAV followed MAP closely but with marked variability between the different animals. The ICC relating TAV to MAP was 0.88 (95% CI. 0.70–0.96) for the animals and 0.94 (95% CI. 0.85–0.98) within the animals. Thus, given the same sequence and animal, there was little residual variation (i.e., TAV robustly predicted MAP well).^29^

### Reverse carotid flow during VCO

A high-resistive triphasic waveform^30^ was present in all VCO sequences. The mean end-diastolic velocity was −29 cm/s at the end of the VCO. Reverse flow started shortly after the initiation of VCO and lasted throughout the whole sequence before returning to baseline (Fig. 1 and Supplemental Fig. 3).

### True PEA

**Fig. 4:**
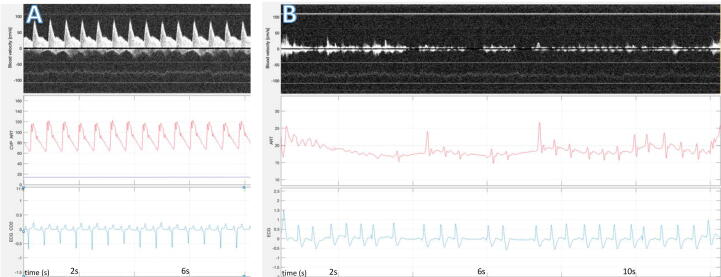
True pulseless electrical activity (PEA). (A) shows baseline before myocardial infarction, B shows true PEA after myocardial infarction. Common to (A) and (B): Top line: RescueDoppler velocity curve, low-velocity flow waveform. Middle line: Arterial pressure, red curve, and central venous pressure, blue curve. Bottom line: electrocardiogram. (A) shows baseline velocity, pressure and ECG. (B) shows true PEA with organized electrical activity on the ECG, arterial blood pressure shows sytolic pressure around 20 mmHg, carotid blood vises aphasic or phasic velocity around 10 cm/s.

After MI, true PEA was identified in two animals. ECG presented with organized rhythm, blood pressure at 20 mmHg and aphasic blood flow velocity (Fig. 4).

## Discussion

This animal study has shown that a novel hands-free carotid Doppler system, RescueDoppler, reliably recorded pulsative flow with blood pressures below 60 mmHg and precisely identified the presence or absence of spontaneous circulation.

The carotid artery is recommended as the gold standard localization for pulse checks as a marker of circulation during CPR.^31^, ^32^ Pulse palpation during cardiac arrest is an unreliable technique.^6^, ^33^, ^34^ Only 38% of healthcare personnel could correctly identify the presence or absence of the carotid pulse within the recommended maximum of 10 seconds.^2^ In a cardiac surgery study where the patients had a systolic pressure greater than 80 mmHg, 45% of the participants could not identify a present pulse.^3^

The prevalence of PEA IHCA is shown to be approximately 35–40% and 22–30% for OHCA.^35^ However, PEA can be misdiagnosed since pulse palpation is unreliable. As many as 32% have been reported to be false PEA^7^ and the prevalence of PEA is increasing.^36^ A correct and early diagnosis is essential to immediately treat the patient's condition to warrant the greatest chance of survival. In our myocardial infarction model, true PEA could be identified with the RescueDoppler showing no blood flow velocity corresponding with no invasive carotid pressure, albeit only in two animals.

Correctly determining whether a patient has achieved ROSC is equally important.^1^ Experimental studies have shown that chest compressions on ROSC may be detrimental to hemodynamics^37^ and unnecessary medication.^15^

Therefore, there is a need for a more reliable method than pulse palpation. The method needs to be non-invasive, continuous and hands-free for identifying circulation during CPR.

In our VCO model, the blood pressures in all sequences were below systolic blood pressure of 60 mmHg. The RescueDoppler recorded continuous pulsative flow in all sequences and might therefore become an alternative to manual pulse palpation.

Several methods to differentiate between pulse or no pulse during resuscitation have been tried, for example, wrist-mounted smartwatches with photoplethysmography that could detect ROSC with the same sensitivity but with a higher specificity than pulse palpation.^38^ Examining the carotid artery during CPR using handheld ultrasound devices is feasible for hemodynamic measurement.^39^, ^40^, ^41^, ^42^, ^43^ B-mode ultrasound-carotid artery compression has been used to assess the carotid artery compressibility and pulsatility by probe compression. The method was quick to determine pulse but limited to the rhythm check time.^40^, ^44^ Detection of ROSC in the femoral artery using a handheld ultrasound was found to be more accurate than pulse palpation.^45^, ^46^

Echocardiography is today recommended in the guidelines but cannot be performed continuously.^47^ Studies have shown that echocardiography nearly doubles the 10 seconds recommended for pulse checks.^1^, ^17^ Echocardiography is also dependent upon image quality and a skilled operator.

Ultrasound measurement of common carotid artery blood flow during CPR is feasible,^43^ where colour flow and spectral Doppler waveform ultrasound images were obtained from the common carotid artery during CPR for 5–10 minutes using a handheld 10-MHz linear array transducer. However, with a handheld probe, continuous measurement is only feasible with a dedicated operator. In a study involving healthy volunteers in a simulated haemorrhage model, systolic blood pressure was reduced to 70 mmHg. A strong correlation was observed between noninvasive stroke volume, carotid artery velocity time integral, and corrected flow time.^19^ No correlation was found between stroke volume change and MAP.^19^ In another study with patients undergoing coronary artery bypass grafting surgery, carotid artery blood flow correlated moderately with invasive cardiac output measurements but less well in tracking changes in cardiac output.^48^ Our study found the strongest correlation within animals between TAV and MAP in the VCO model with an ICC of 0.94.

Development of hands-free, carotid Doppler is in progress and seems promising for indicating blood circulation during resuscitation. However, these systems use continuous wave doppler, which cannot define the depth of the velocities.^49^ With pulse wave Doppler, it is possible to identify blood velocities and flow direction in a specific depth, from arteries and veins, respectively.^19^, ^27^, ^50^

A hands-free carotid continuous wave Doppler system has previously been studied by Larabee et al. in a swine model where carotid flow velocity was detected over a wide range of blood pressures.^27^ Their system could differentiate pseudo from true PEA during CPR in cardiac arrest and detect pressure gradient changes of less than 5 mmHg through to normotension. RescueDoppler uses pulsed wave Doppler that measures blood velocity at a precise location and differentiates flow and direction between arteries and veins, respectively. RescueDoppler is limited in its ability to quantify the amount of flow, but the graphic display of velocities over time and colour M-mode provides the ability to interpret information with less subjectivity than with colour Doppler alone.^51^, ^52^, ^53^

In our VF model, the RescueDoppler recorded the absence of flow immediately with the cessation of invasive blood pressure in all animals and sequences. We did not observe any significant impact on the placement of the device or Doppler signals due to the movements resulting from defibrillation. The potential of RescueDoppler to detect ROSC during or in between chest compressions will be further investigated. Also, identifying circulation in special circumstances like cardiac arrest in severe accidental hypothermia is crucialy.^16^ Identifying blood flow and trends can provide valuable insights into the progression and treatment. We did not investigate hypothermia in our study, but this is in the scope for future studies.

## Limitations

Despite similarities between human and porcine hemodynamics, there are still species-based differences. In pigs, the musculature of the head and neck and large mass of soft tissue is supplied by the robust external carotid arteria. Only a tiny ascending pharyngeal artery leads to the internal carotid artery distal to the rete mirable^54^. The anatomical differences from humans are believed to entail high resistance triphasic flow in the common carotid artery in the VCO model. These high-resistance triphasic flow findings are not immediately transferable to humans and must be investigated further in clinical trials. The sensitivity of the RescueDoppler to head rotation and neck extension during resuscitation procedures represents an important factor that warrants further clinical investigation.

These differences mean porcine-based findings, especially the Doppler tracings, might not be directly transferable to human medicine. However, the differences between pigs and humans do not affect the operational properties of RescueDoppler, and we consider our findings to be transferable to human medicine, albeit with a human calibration.

## Conclusion

This animal study has shown that a novel hands-free pulsed wave continuous carotid Doppler system, RescueDoppler, reliably identified pulsative flow with blood pressures below 60 mmHg. RescueDoppler has the potential of replacing unreliable pulse palpation during CPR. Blood flow cessation was promptly and accurately identified during VF and in two animals with PEA during myocardial infarction.

## CRediT authorship contribution statement

**Bjørn Ove Faldaas:** Conceptualization, Formal analysis, Software, Investigation, Writing – original draft, Visualization. **Erik Waage Nielsen:** Conceptualization, Methodology, Software, Validation, Investigation, Writing – review & editing. **Benjamin Stage Storm:** Conceptualization, Validation, Investigation, Data curation, Writing – review & editing. **Knut Tore Lappegård:** Methodology, Validation, Investigation, Writing – review & editing. **Ole-Jakob How:** Methodology, Validation, Investigation, Writing – review & editing. **Bent Aksel Nilsen:** Investigation, Resources, Writing – review & editing. **Gabriel Kiss:** Software, Data curation, Writing – review & editing. **Eirik Skogvoll:** Conceptualization, Methodology, Validation, Formal analysis, Writing – review & editing, Visualization. **Hans Torp:** Conceptualization, Methodology, Software, Validation, Investigation, Data curation, Writing – review & editing. **Charlotte Ingul:** Conceptualization, Methodology, Validation, Formal analysis, Investigation, Writing – review & editing, Supervision, Project administration.

## Declaration of Competing Interest

Hans Torp and Charlotte Björk Ingul are employed by Cimon Medical, which owns the technology associated with RescueDoppler. Hans Torp has stocks in Cimon Medical. Knut Tore Lappegård has received a lecture honorarium from Boehringer Ingelheim Gmbh without relation to the present study.
