# Supplementary material for: Hands-free continuous carotid Doppler ultrasound for detection of the pulse during cardiac arrest in a porcine model
Source: Resusc Plus. 2023 Jun 20;15:100412. doi: 10.1016/j.resplu.2023.100412 (PMC10336194; doi:10.1016/j.resplu.2023.100412)
Supplement: Supplementary Fig. 1 [file mmc1.pdf]

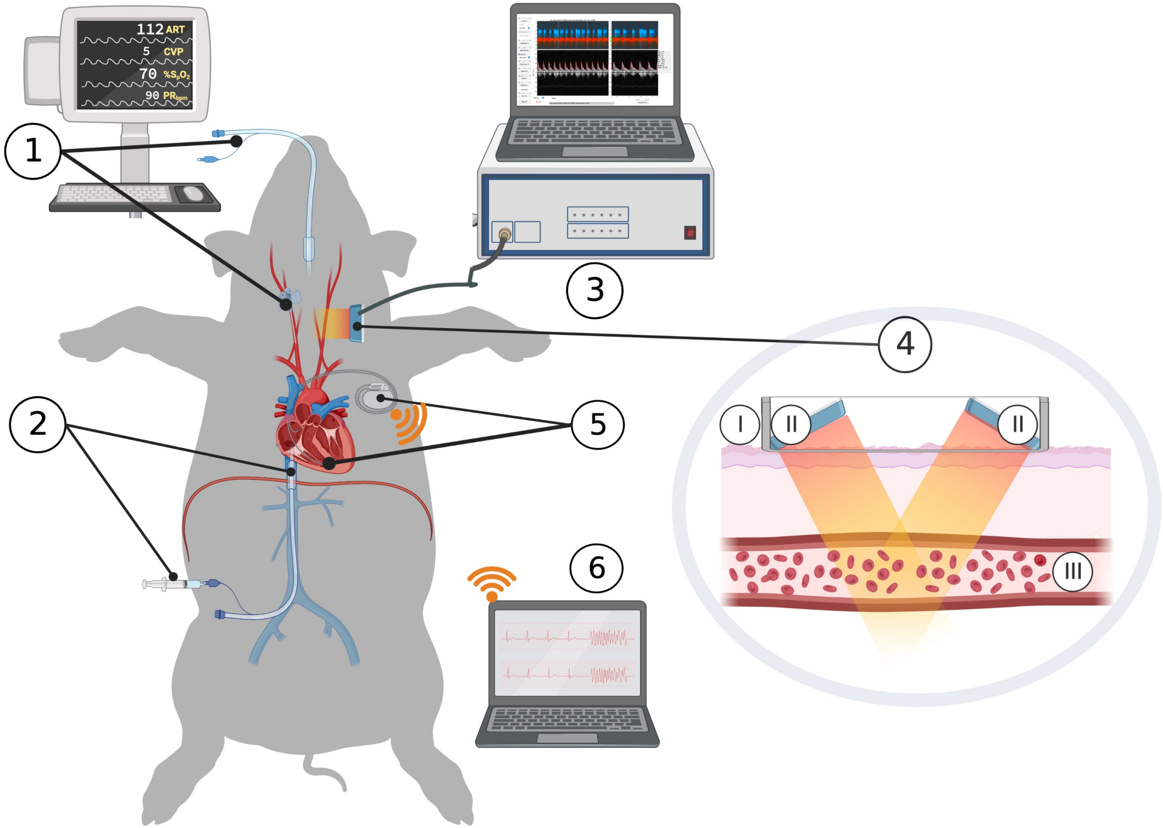


Supplement figure 1. RescueDoppler research setup: 1) A patient monitor recorded continuous 5-lead electrocardiogram, continuous invasive arterial- and central-venous pressures, EtCO2, SpO2, and urinary bladder temperature; 2) The vena cava was occluded by inflation of an Edwards Fogarty Arterial Embolectomy Catheter with the tip in the diaphragmatic level of the inferior vena cava. 3) The RescueDoppler probe was connected to a Manus EIM-A scanner and a computer running a custom Matlab program to record and display color M-mode and Doppler spectrogram. 4) The RescueDoppler probe was attached to the neck, perpendicular to the carotid artery over the carotid artery. Insert: Illustration of transducer prototype setup in detail: I) 3D printed holder for two transducers with a fixed +/- 30° angle. II) Transducers, four MHz with a size of 30 x 6 mm., depth range 8 to 45 mm. III) Carotid Artery with red blood cells 5). St. Jude Medial Ellipse DR ICD with ICD lines placed in the right ventricle apex. 6) Merlin Patient System for control of the implanted IDC. The figure was created using Biorender.com.
