# Supplementary material for: Hands-free continuous carotid Doppler ultrasound for detection of the pulse during cardiac arrest in a porcine model
Source: Resusc Plus. 2023 Jun 20;15:100412. doi: 10.1016/j.resplu.2023.100412 (PMC10336194; doi:10.1016/j.resplu.2023.100412)
Supplement: Supplementary Fig. 2 [file mmc2.pdf]

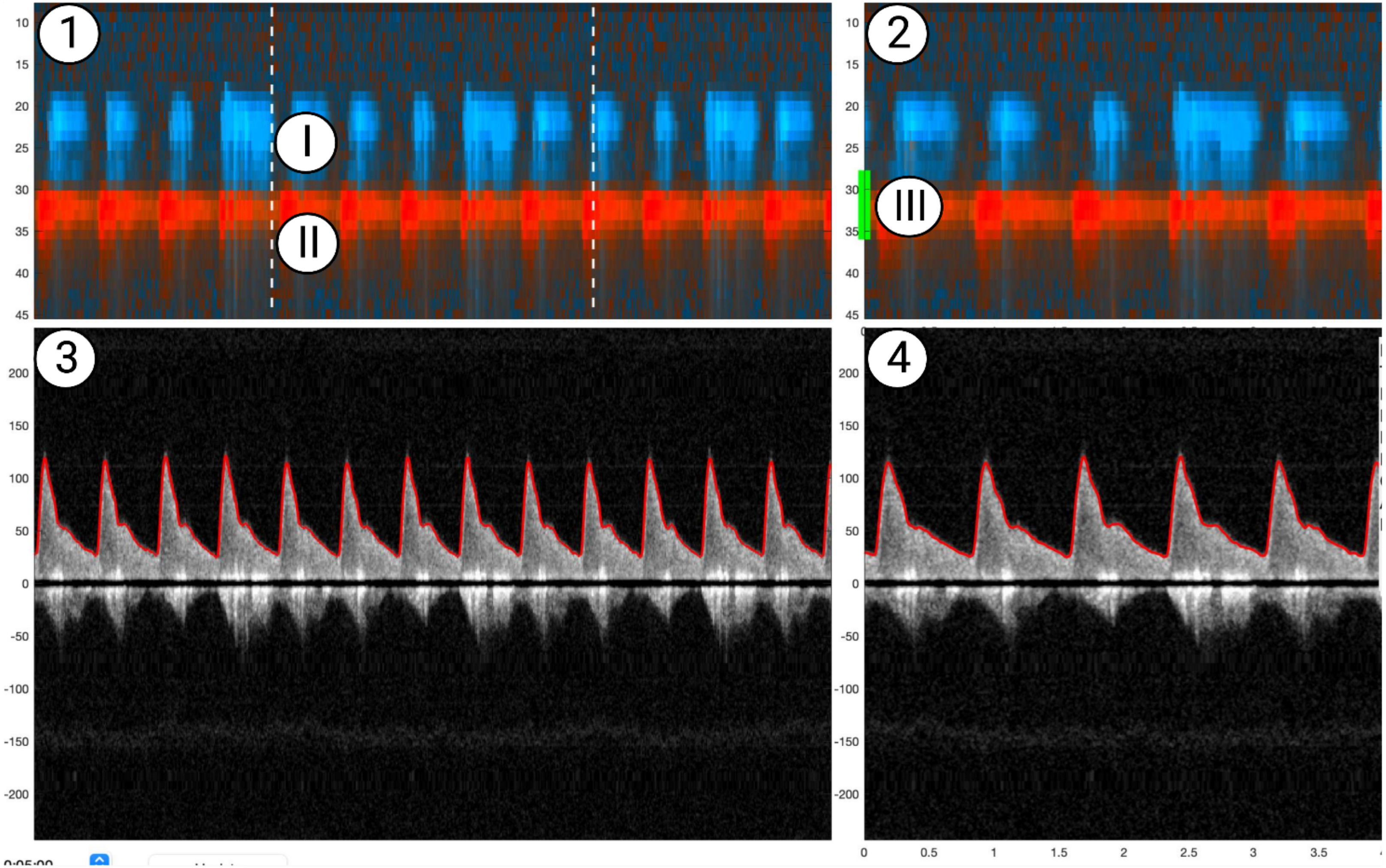

Supplement figure 2
Matlab specter curve in upper two panels (1, 2), depth-vs-time color M-mode. Velocities (blood flow) from several vessels at different depths between 8 and 45 mm. 1) Left upper panel shows the total time recorded, 2) right upper panels shown an adjustable duration from 1-4 s. 3 and 4 shows Doppler specter flow curve. I)Blue color indicating blood flow moving from the brain, II) red color indicating blood moving towards the brain. III)Green bar indicating sample volume depth and volume. 4) Doppler velocity curve from sample volume selected above.
