# Supplementary material for: Hands-free continuous carotid Doppler ultrasound for detection of the pulse during cardiac arrest in a porcine model
Source: Resusc Plus. 2023 Jun 20;15:100412. doi: 10.1016/j.resplu.2023.100412 (PMC10336194; doi:10.1016/j.resplu.2023.100412)
Supplement: Supplementary Fig. 3 [file mmc3.pdf]

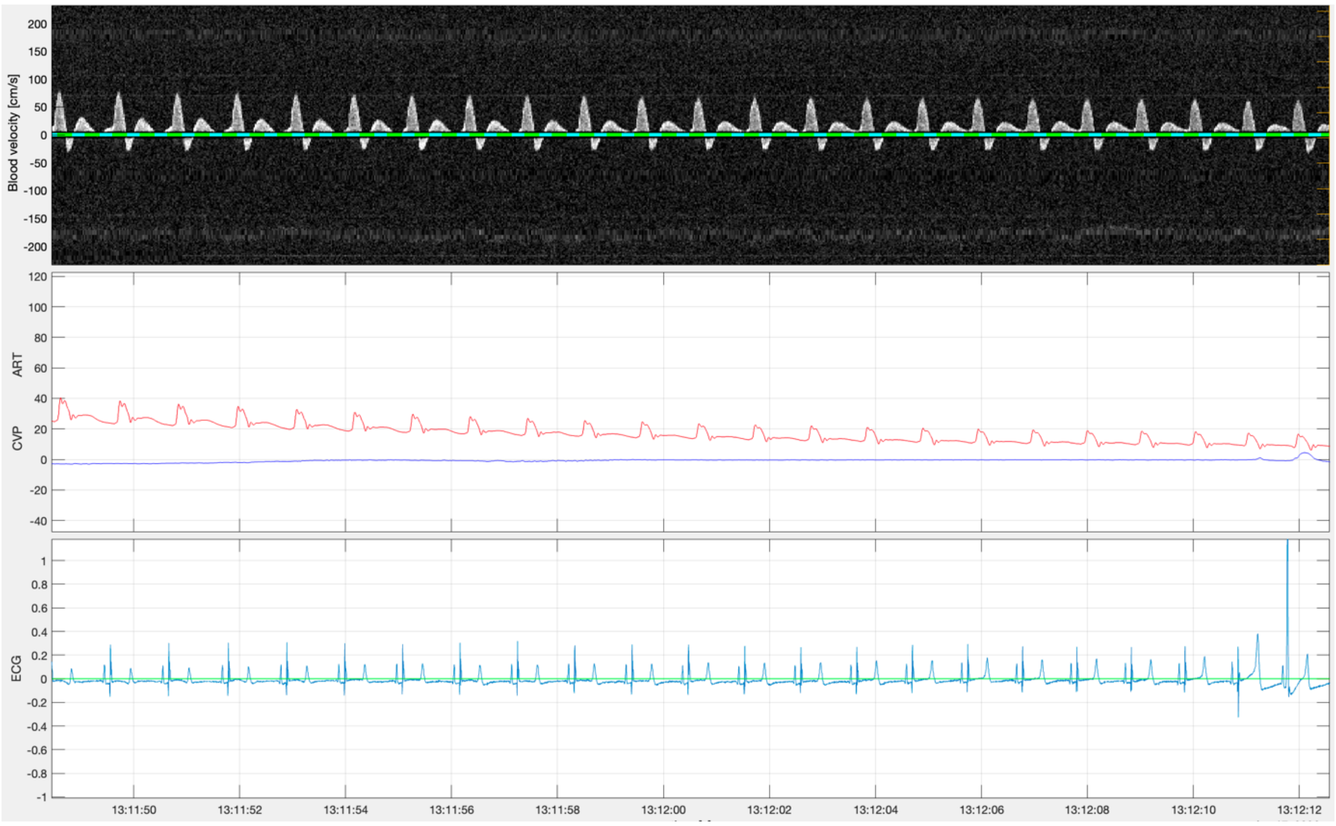

Supplement figure 4

Last 24s of a VCO sequence. Top line: RescueDoppler velocity curve, triphasic flow waveform. Middle line: Arterial pressure, red curve, and central venous pressure, blue curve. Bottom line: ECG. This example shows the last part of a VCO sequence with blood pressure at systolic 40 mm Hg and fall to the lowest of 19 mm Hg before occlusion ceases. Doppler peak flow falls from 70 to 60 cm/s.
