# Supplementary material for: Hands-free continuous carotid Doppler ultrasound for detection of the pulse during cardiac arrest in a porcine model
Source: Resusc Plus. 2023 Jun 20;15:100412. doi: 10.1016/j.resplu.2023.100412 (PMC10336194; doi:10.1016/j.resplu.2023.100412)
Supplement: Supplementary Table 1 [file mmc4.pdf]

**Supplemental Table 1**

Overview of data recorded from the monitor, ventilator, and RescueDoppler system. Data was synchronized in a custom Matlab program after the experiments.

| |
|---|
| Phillips MP70 |
| Electrocardiogram |
| Invasive systolic blood pressure |
| Invasive diastolic blood pressure |
| Mean Arterial Pressure |
| End-tidal carbon dioxide |
| fraction of inspired oxygen |
| Oxygen saturation |
| Temperature |
| Respirator |
| Respiratory rate |
| Tidal volume |
| Positive end-expiratory pressure |
| Doppler scanner |
| Peak systolic velocity |
| Time Averaged Velocity |
| End Diastolic Velocity |
| Heart Rate |
| Pulsative Index |
| Resistance index |
